# Are conspiracy theorists psychotic? A comparison between conspiracy theories and paranoid delusions

**DOI:** 10.1192/j.eurpsy.2022.2059

**Published:** 2022-09-01

**Authors:** W. Veling, B. Sizoo, J. Van Buuren, C. Van Den Berg, W. Sewbalak, G. Pijnenborg, N. Boonstra, S. Castelein, L. Van Der Meer

**Affiliations:** 1 University Medical Center Groningen, University Center For Psychiatry, Groningen, Netherlands; 2 Dutch National Police, National Unit, Driebergen, Netherlands; 3 Leiden University, Institute Of Security And Global Affairs, Den Haag, Netherlands; 4 GGz Delfland, Poli Schiedam, Schiedam, Netherlands; 5 GGZ Drenthe, Department Of Psychotic Disorders, Assen, Netherlands; 6 University of Groningen, Clinical And Developmental Neuropsychology, Groningen, Netherlands; 7 University of Applied Sciences, Care And Innovation, Leeuwarden, Netherlands; 8 Lentis Psychiatric Institute, Lentis Research, Groningen, Netherlands; 9 University of Groningen, Experimental Psychopathology And Clinical Psychology, Groningen, Netherlands; 10 Lentis Psychiatric Institute, Department Of Rehabilitation, Zuidlaren, Netherlands

**Keywords:** conspiracy theories, paranoid delusions, Covid-19

## Abstract

**Introduction:**

Conspiracy theories are popular during the COVID-19 pandemic. Conspiratorial thinking is characterised by the strong conviction that a certain situation that one sees as unjust is the result of a deliberate conspiracy of a group of people with bad intentions. Conspiratorial thinking appears to have many similarities with paranoid delusions.

**Objectives:**

To explore the nature, consequences, and social-psychological dimensions of conspiratorial thinking, and describe similarities and differences with paranoid delusions.

**Methods:**

Critically assessing relevant literature about conspiratorial thinking and paranoid delusions.

**Results:**

Conspiratorial thinking meets epistemic, existential, and social needs. It provides clarity in uncertain times and connection with an in-group of like-minded people. Both conspiratorial thinking and paranoid delusions involve an unjust, persistent, and sometimes bizarre conviction. Unlike conspiracy theorists, people with a paranoid delusion are almost always the only target of the presumed conspiracy, and they usually stand alone in their conviction. Furthermore, conspiracy theories are not based as much on unusual experiences of their inner self, reality, or interpersonal contacts.

**Conclusions:**

Conspirational thinking is common in uncertain circumstances. It gives grip, certainty, moral superiority and social support. Extreme conspirational thinking seems to fit current psychiatric definitions of paranoid delusions, but there are also important differences. To make a distinction with regard to conspiratorial thinking, deepening of conventional definitions of delusions is required. Instead of the strong focus on the erroneous content of delusions, more attention should be given to the underlying idiosyncratic, changed way of experiencing reality.

**Disclosure:**

No significant relationships.

